# Selection of Anti-Sulfadimidine Specific ScFvs from a Hybridoma Cell by Eukaryotic Ribosome Display

**DOI:** 10.1371/journal.pone.0006427

**Published:** 2009-07-29

**Authors:** Yonghua Qi, Congming Wu, Suxia Zhang, Zhanhui Wang, Siyang Huang, Lei Dai, Shaochen Wang, Lining Xia, Kai Wen, Xingyuan Cao, Yongning Wu, Jianzhong Shen

**Affiliations:** 1 National Center for Veterinary Drug Safety Evaluation, College of Veterinary Medicine, China Agricultural University, Beijing, China; 2 College of Animal Science, Henan Institute of Science and Technology, Xixiang, China; 3 College of Veterinary Medicine, Xinjiang Agricultural University, Urmuqi, China; 4 Institute for Nutrition and Food Safety, Chinese Center for Disease Control and Prevention, Beijing, China; University of Southampron, United Kingdom

## Abstract

**Background:**

Ribosome display technology has provided an alternative platform technology for the development of novel low-cost antibody based on evaluating antibiotics derived residues in food matrixes.

**Methodology/Principal Findings:**

In our current studies, the single chain variable fragments (scFvs) were selected from hybridoma cell lines against sulfadimidine (SM_2_) by using a ribosome library technology. A DNA library of scFv antibody fragments was constructed for ribosome display, and then mRNA–ribosome–antibody (MRA) complexes were produced by a rabbit reticulocyte lysate system. The synthetic sulfadimidine-ovalbumin (SM_2_-OVA) was used as an antigen to pan MRA complexes and putative scFv-encoding genes were recovered by RT-PCR *in situ* following each panning. After four rounds of ribosome display, the expression vector pCANTAB5E containing the selected specific scFv DNA was constructed and transformed into *Escherichia coli* HB2151. Three positive clones (SAS14, SAS68 and SAS71) were screened from 100 clones and had higher antibody activity and specificity to SM_2_ by indirect ELISA. The three specific soluble scFvs were identified to be the same molecular weight (approximately 30 kDa) by Western-blotting analysis using anti-E tag antibodies, but they had different amino acids sequence by sequence analysis.

**Conclusions/Significance:**

The selection of anti-SM_2_ specific scFv by *in vitro* ribosome display technology will have an important significance for the development of novel immunodetection strategies for residual veterinary drugs.

## Introduction

Sulfadimidine, derivatives of ρ-aminobenzenesulfonamide, is widely used in veterinary and human medicine for prophylactic and therapeutic purposes. It is also used as additive of animal feed due to their growth promotion properties. However, the proper withdrawal periods need to be done before slaughtering or milking in the medicated animals. Otherwise the meat and milk from these animals may be contaminated with residual SM_2_, leading to adverse effects (toxic action and resistance) in human. In the USA, European Union and Canada, the maximum residue limit (MRL) of total sulfonamides in edible tissues is 100 µg/kg, and 20 µg/kg in Japan [1]–[3].

The monitoring programs, especially immunochemical screening methods have been widely used to evaluate antibiotics derived residues in food matrixes. Current conventional methods for the analysis of sulfonamides derived residue are microbiological tests and analytical methods, such as thin-layer chromatography or high-performance liquid chromatography. However, these methods require well equipped laboratory, trained personnels, high capital expenditure and time-consuming sample preparation steps. Immunochemical assays such as enzyme linked-immunosorbent assay (ELISA) are simple, rapid, sensitive, specific, and generally cost-effective for large sample loads[4]. A number of immunochemical assays have been developed to screen sulfonamide [5]–[7]. However, Current sulfonamides immunochemical assays use conventional polyclonal (PAb) and monoclonal antibodies (MAb). PAbs are the easiest and quickest to produce, but they are not single molecular entities and sometimes cause nonspecific reactivity. MAbs are single molecular entities, and multiple clones are available for selection in the development process, but the preparation of MAb is more complex, and expensive cell culturing facilities are required for large scale production [8].

Recently, recombinant antibody display technology has provided an alternative platform technology for the development of novel low-cost antibody based biotherapeutics and biological detection [9], [10]. One of the most remarkable molecules of recombinant antibodies is the single chain variable fragment (scFv), which is made by connecting the variable heavy chain with light chain region. This structure still retains the binding properties of classical antibody. ScFv technology is a new strategy for developing improved immunodetection tests for veterinary drugs [11], [12]. ScFv antibodies can be generated by phage display or ribosome display technologies. Although phage display represents a considerable progress compared to hybridoma technology, it is still not a perfect technique. First, the necessary transformation step limits the library size. Secondly, the selection in the context of the host environment cannot be avoided and their growth disadvantage or toxicity for *Escherichia coli* possibly lead to a loss of potential candidates. Furthermore, difficulties in eluting phages carrying antibodies with very high affinity may be encountered [13], [14]. Ribosome display, created by Mattheakis et al and modified by Hanes and Plückthun as well as He and Taussig, is a robust tool for the isolation of specifically binding antibody fragments and non-immunoglobulin scaffolds [15]–[21]. It is based on the formation of a mRNA-Ribosome-Antibody(MRA) ternary complexs during *in vitro* expression. In the ribosome display, those of the limitations of phage display are circumvented by utilizing a cell-free transcription, translation and panning system. A larger capacity and further diversity of libraries will be built up and the random mutations can be introduced by PCR. It has exceptional strength in molecular evolution and affinity maturation. By using this novel technology, it is currently possible to select and evolve the high-affinity antibodies [20], [22], [23].

In this study, we hypothesize that scFvs specific for anti-sulfadimidine from a hybridoma cell can be produced and the affinity-matured efficiently using ribosome display technology and envisage that these unique scFvs will be valuable diagnostics in agriculture and the food industry. We hope that this study would provide a pathway for the development of a novel immunoassay on residual SM_2_ detection by using recombinant antibody.

## Results and Discussion

### Antibody library construction

**Figure 1:**
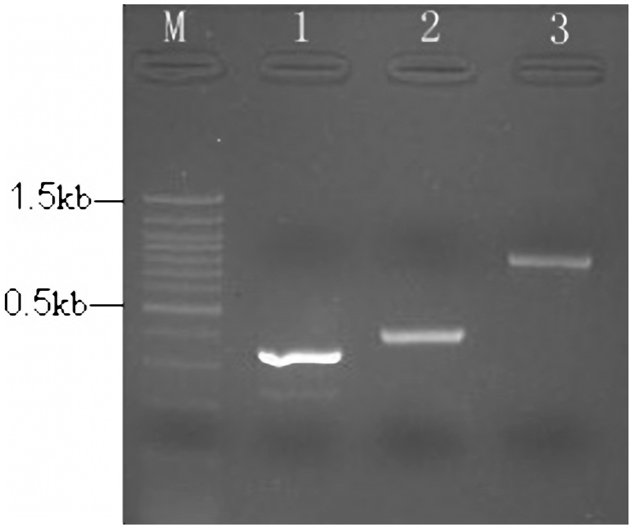
Agarose gel electrophoresis of amplified V_H_ chain, V_L_ chain and assembled scFv fragments. Lane M: 100 bp plus DNA marker, lane 1: V_H_ fragments, lane 2: V_L_ fragments, lane3: about 800 bp assembled scFv fragments.

V_H_ and V_L_ fragments were amplified by RT-PCR from hybridoma cell lines secreting anti-SM_2_ MAb and assembled into full-length scFvs library with the (Gly_4_Ser)_3_-linker sequence. The amplified V_H_ and V_L_ fragments were the expected size (about 340 bp and 325 bp). **(** **Fig. 1** **)**. The assembled approximate 0.8 kb full-length scFv fragments were used for the construction of templates of ribosome display.

### Ribosome display and in situ RT-PCR recovery scFv

**Figure 2:**
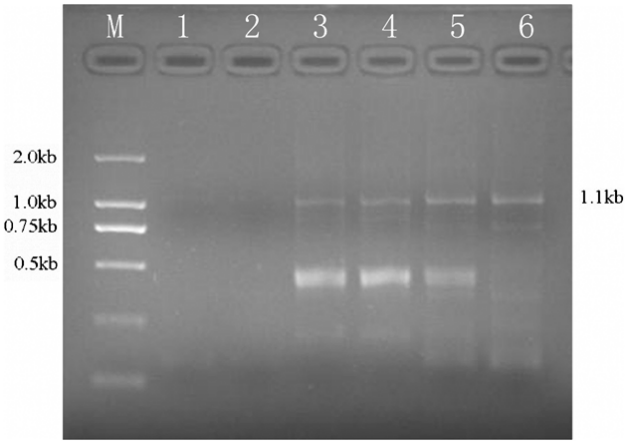
Selection and amplification of anti-SM_2_ scFv gene over four rounds of ribosome display. After selection, the selected product was amplified by SP-PCR and the SP-PCR products were analyzed by agarose gel electrophoresis. lane M: DL2000 DNA marker, lane 1: preliminary translation mixture selected on a PBS-coated well, lane 2: translation mixture selected on a OVA-coated well, lane 3: recovered band from the first ribosome display, lane 4: recovered band from the second round, lane 5: recovered band from the third cycle, lane 6: recovered band from the fourth selected library.

The gel-purified scFv fragments were digested and ligated into the vector pRDV. The ligation product was directly used as a template for the amplification of initial ribosome display library. The original scFv library was subjected to *in vitro* transcription and translation using **TNT** T7 Quick for PCR DNA kit to generate ternary MRA complexes. The target antigens were immobilized on microtiter plates. The selected specific scFv fragment based on mRNA of retained MRA complexes bound to SM_2_-OVA at the plate wells was recovered after several washing with increasing stringency during the individual rounds of selection by in situ RT-PCR and SP-PCR. The obtained products were applied to the affinity maturation or next round of panning. Each cycle of ribosome display was performed under the same conditions including the concentration of target antigens and the spanning washing time. As a whole, four cycles of selection on SM_2_-OVA, as well as one round of affinity maturation were performed. The panning progress was monitored by examining the intensity of SP-PCR products (approximately 1.1 kb) on agarose gel–electrophoresis. The quantity of SP-PCR products continually increased during the next rounds of panning. Based on the result of SP-PCR, enrichment of specific scFvs was clearly confirmed. Meanwhile, no band was observed PBS-coated wells or when the untranslated mixture was used. **(** **Fig. 2** **)**.

Here, we performed eukaryotic ribosome display by using an *in vitro* coupled transcription/translation system. These processes, based on the *E.coli.S30* and rabbit reticulocyte systems, had been described in previous studies [19], [24]–[29]. We carried out the coupled transcription/translation steps, which were conducted by using a rabbit reticulocyte system. Briefly, after the transcription process, the translation was directly completed in tandem, potentially resulting in greater functional expression of scFvs antibodies [25]. Meanwhile, the rabbit reticulocyte system has lower RNase activity than an *E.coli.S*30 ribosome display system, leading to a less complicated selection condition [26]. Moreover, eukaryotic conditions may improve the translation or folding efficiency of some proteins. Then in our work, the eukaryotic ribosome display with coupled transcription/translation was used for selection and evolution of specific antibody.

In addition, the principal distinction between the *E.coli.S*30 and rabbit reticulocyte systems is in the DNA recovery step. In the *E.coli.S*30 system, a chemical disruption procedure (EDTA) is introduced to dissociate the ribosome and release the mRNA, which is then purified before RT-PCR. Although this procedure efficiently isolates mRNA from *E.coli* ribosome complexes, it gives a relatively poor recovery from those generated by rabbit reticulocyte lysate [25], [27], so that it cannot be applied directly to eukaryotic MRA complexes. We introduced the in situ RT-PCR procedure in which an internal primer is used for performing reverse transcription directly on the eukaryotic ribosome complexes without their disruption or mRNA isolation [28], [29]. Not only does this simplify the recovery process, but it also avoids losses incurred in complex disruption. This procedure has been proved to be efficient and reliable in cDNA recovery in other studies [25], [28], [30]. The ineffectiveness, in the in situ procedure, of a primer that recognizes the 3′ end of the mRNA, compared with the efficient use of one hybridizing upstream, is consistent with the interpretation that the ribosome is stalled at the end of the mRNA, which is consequently unavailable at the initiation of reverse transcription. The possibility of the 3′ end of the mRNA being degraded, as has been speculated, has been excluded by showing that mRNA released from the ribosome complexes by EDTA, although low in yield, could be recovered and amplified by the 3′ terminal primer with similar efficiency to the internal primer. The single-primer RT-PCR procedure presented here is a refinement of the in situ DNA recovery method. It is based on the finding that single-primer PCR technology is capable of amplifying efficiently individual molecules of dsDNA fragments carrying identical flanking sequences at each end [31]. We have adapted this concept to in situ RT-PCR recovery by producing single-stranded cDNAs with complementary flanking 5′ and 3′ terminal sequences, so that PCR can be performed using a single consensus primer (KZ) to amplify the resultant cDNA templates. The method involves a new reverse transcription primer (RT-kz), the 3′ end of which hybridizes ∼60 nt upstream of the 3′ terminus of the mRNA, and the 5′ end includes a consensus sequence from the transcription start to the translation initiation site. The in situ recovery method, using RT-PCR on an mRNA template that has not been prereleased from the ribosome, provides high sensitivity in a simple procedure and avoids sample loss.

### The Characterization of soluble scFv

**Figure 3:**
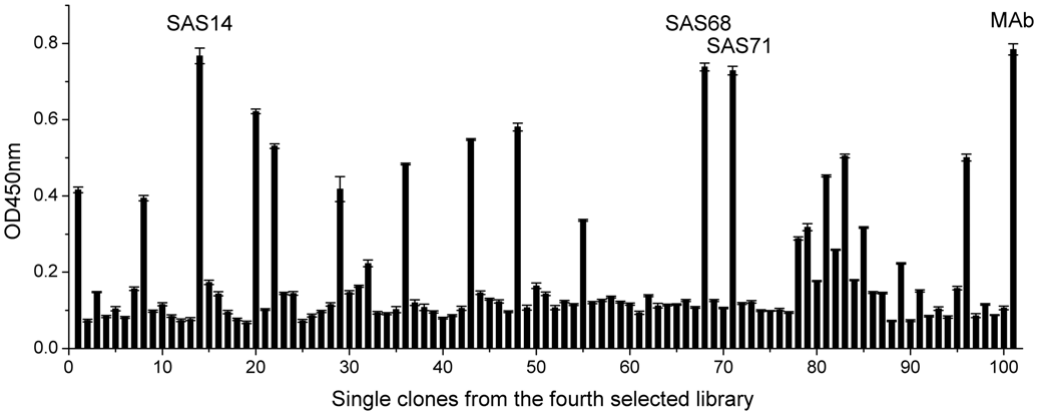
Clones were isolated from the fourth selected antibody library. Each clone was expressed for producing soluble scFv, and the binding activity of each scFv and the anti-SM2 MAb (positive control) was determined by ELISA (in triplicate). The error bars represent the standard deviation.

**Figure 4:**
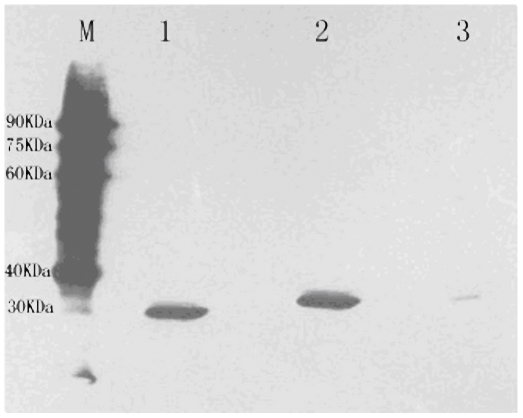
Detection of soluble scFvs in periplasmatic extracts by Western Blot. M: low molecular weight protein marker, lanes1–3: Periplasmatic extracts of scFvs SAS14, SAS68, and SAS71.

The selected specific scFv fragment after the fourth round was ligated with the expression vector **pCANTAB5E** for soluble scFvs expression. After electrotransformation, around 100 colonies from the selected library were isolated and soluble proteins of these clones were expressed using the nonsuppressor strain *E.coli* HB2151. The periplasmic extracts from individual clones were tested by indirect ELISA. The result showed that few clones showed positive to SM_2_ in ribosome-ELISA before panning (**Fig. S1**), however, several clones from the fourth selected library had a good conjugation activity to specific SM_2_-OVA. Among these clones, the three clones (SAS14, SAS68, SAS71) exhibiting the highest ELISA signals to SM_2_-OVA were not significantly different to the anti-SM_2_ MAb (**Fig. 3** **)**. The result suggested that the conjugation activity of scFvs was similar to that of parent MAb. The three specific soluble scFvs were confirmed by SDS-PAGE and Western-blotting analysis using anti-E tag antibodies. Approximately 30 kDa scFv protein was expressed from each of the three selected clones in the periplasmic extract sample compared to negative control (**Fig. 4**).

**Figure 5:**
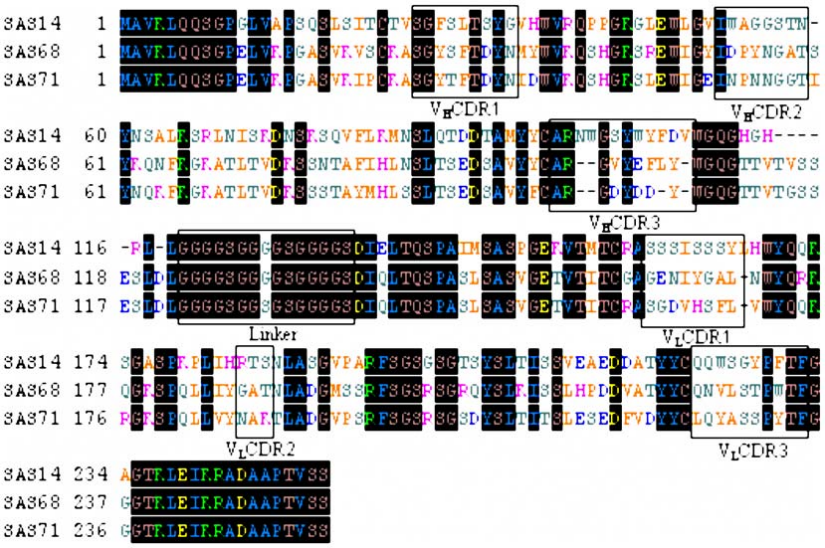
Alignment of the amino acid sequence of selected specific anti-SM_2_ scFvs. CDRs and the (Gly_4_Ser)_3_ linker are boxed. The regions of CDR1-CDR3 were deduced according to Kabat database.

The deduced amino acid sequences of the above-mentioned three scFvs and complementary determining regions (**CDR**) were shown in **Fig. 5**. By using the DNA sequences, the V_H_ and V_L_ gene families of the scFvs were designated based on Werner Müller's database (**DNAPLOT** software). The heavy chains of scFvs SAS68 and SAS71 belong to the V_H_1 gene family and that of SAS14 belongs to the V_H_2 gene family. The light chains of clones SAS68 and SAS71 belong to the Vk IGKV12/13 subgroup and that of SAS14 belongs to the Vk IGKV4/5 subgroup. Sequencing alignment by the Vector **NTI** software program showed that the V_H_ of the clones SAS68 and SAS71 have very similar sequences (90.44% homology) and the V_L_ of SAS14, SAS68 and SAS71 shared 82.81% homology. Although the linking sequence of scFv SAS71 showed some mutations, these did not influence protein structure obviously. This indicated that a random mutation was induced by the second ribosome display, but mutation affected scFv structures were selected. Sequencing data indicated that we succeeded in achieving our desired goals of library generation with the affinity maturation method.

*In vitro* selection cycles enable the introduction of further diversity every iteration, providing a means of protein evolution. Thus, ribosome display and similar methods are routes to increased diversity, and improve selection and a range of potentially novel molecules [32]. We carried out the affinity maturation by using error-prone PCR (EP-PCR) and staggered extension process (StEP) shuffling in tandem during the second round of ribosome display. The enrichment of specific scFvs was confirmed by the SP-PCR recovery band from each ribosome display cycle and the binding activity of scFvs from the fourth selected antibody library, respectively. After four rounds of ribosome display, three scFvs with high specificity and affinity were successfully selected. It showed that the slightly modified ribosome display technology was feasible for selection of specific scFvs against SM_2_.

### Conclusions

In this report, we demonstrated the generation of anti-SM_2_ specific scFvs from hybridoma cell lines by eukaryotic ribosome display. Since ribosome display technology was first reported in 1994 [18], some early studies suggested antibody selection from a library using ribosome display[25], [28], [29], [32], [33]. However, there have been few reported about selection of scFvs against SM_2_. Therefore, our work is significant to be an example for selecting specific scFvs against veterinary drugs by eukaryotic ribosome display. As an alternative to antiserum or MAb, the selected specific scFvs with high affinity can be used as detection reagent of SM_2_ in foodstuffs in the future. In addition, this work provides a novel pathway for the development of a rapid, sensitive and multi-residue immunoassay analysis technology for veterinary drugs detection with using recombinant antibody.

## Materials and Methods

### Plasmids, Strains and Reagents

All reagents used in the study were commercially available and were of reagent grade or better. All restriction enzymes and DNA modification enzymes were of molecular biology grade. DH5α and pGEM-T easy cloning vectors were purchased from TaKaRa; The ribosome display vector (pRDV) was obtained as a gift from the lab of Prof. Andreas Plückthun (Biochemisches Institut, Universität Zürich, Switzerland) [16]; The phagemid pCANTAB5E was from Amersham Biosciences; The bacterial host used for cloning and expression was the non-suppressor strain -*E. coli* HB2151, which was a gift from the lab of Prof. Yuanming Sui (Food Quality and Safety Research Institute, South China Agriculture University, P. R. China,); TNT T7 Quick for PCR DNA kit (rabbit reticulocyte cell free extract) was from Promega; Goat anti-E-Tag Antibody Affinity Purified HRP conjugated was purchased from Bethl Laboratories Inc.

### Construction of scFv library

**Figure 6:**
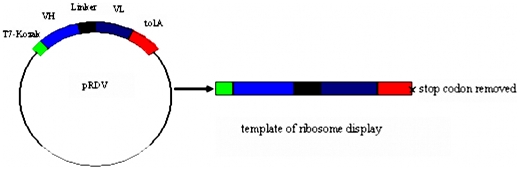
Construction of the template of ribosome display. In this generalized construct for ribosome display, the gene of interest is flanked by a 5′sequence with the T7 promoter (T7) and eukaryotic translation initiation (Kozak) sequence, and a tolA domain is attached at the 3′end as a spacer. The stop codon is removed to ensure stalling of the ribosome at the end of translation. X indicates removal of the stop codon.

Total RNA was extracted from 10^7^ Hybridoma cells produced previously by He et al [34] that secreted monoclonal antibody against SM_2_ by using SV Total RNA Isolation System (Promega, USA). About 0.5 µg of RNA was reverse transcribed by Oligo dT-Adaptor primers of the first-strand cDNA synthesis kit (TaKaRa, JAPAN). cDNA encoding the mouse variable heavy (V_H_) and light (V_L_) chains were amplified by RT-PCR with degenerated immunoglobulin PCR primers (35 cycles at 94°C for 30 s, 55°C for 30 s and 72°C for 1 min). A complementary (Gly_4_Ser)_3_-linker was added by a re-amplification. The full-length scFvs were assembled by using splicing overlap extension PCR. The purified products were cloned into the vector pRDV to add a 5′-T7-promotor and ribosome binding site and 3′-spacer region **(** **Fig. 6** **)**. The templates of ribosome display were amplified directly from the ligation mixture [35]. The primers used in the PCR amplification were shown in **Table 1**.

**Table 1:** Synthesized oligonucleotides for the construction of scFv and ribosome display library.

Primer name	sequence
**V_H_For**	AGATCTAGAGAATTCTGAGGAGACGGTGACCGTGGTCCCTTGGCCCCAG
**V_H_Rev**	AGATCTAGAAAGCTTAGGTCAAGCTGCAGCAGTCAGG
**V_L_For**	GGATACAGTTGGTGCAGCATC
**V_L_Rev**	GACATCCAGCTGACTCAGTCT
**V_H_For** ***NcoI***	CATGCCATGGATGGCCGTCAAGCTGCAGCAGTCAGGA
**V_H_LinkRev**	ACCACCGGATCCGCCTCCGCCTAGATCTAGAGATTCTGAGGAGA
**V_L_LinkFor**	GGAGGCGGATCCGGTGGTGGCGGATCTGGAGGTGGCGGAAGCGACATCCAGCTGACTCAGTC
**V_L_Rev** ***HindIII***	CCCAAGCTTGGATACAGTTGGTGCAGCATC
**T7B-kz**	GCAGCTAATACGACTCACTATAGGAACAGACCACCATGGCCGTCAAGCTGCAGCAG
**tolAk**	CCGCACACCAGTAAGGTGTGCGGTTTCAGTTGCCGCTTTCTTTCT
**RT-kz**	GAACAGACCACCATGCTGCTTCTGCCGCTTCC
**KZ**	GAACAGACCACCATG
**scFvFor** ***SfiI***	GTCCTCGCAACTGCGGCCCAGCCGGCCATGGCCGTCAAGCTGCAGCAGTCAGGA
**scFvRev** ***NotI***	GAGTCATTCTGCGGCCGCGGATACAGTTGGTGCAGCATC

The underlined sequence indicates the *NcoI*, *HindIII*, *SfiI* and *NotI* restriction site for cloning.

### In vitro transcription and translation

Eukaryotic ribosome display was conducted by a modified method, which was reported previously [24], [26], [36]. Briefly, 50 µl of transcription/translation mixture, containing 40 µl TNT T7 quick for PCR mix, 0.02 mM methionine and 1 mM Mg acetate, was set up in a siliconized tube with 100–700 ng purified PCR porducts. After incubation at 30°C for 60 min, the following reagents were added: 60 units DnaseI (TaKaRa, JAPAN), 7 µl 10×DNase I digestion buffer(400 mM Tris/HCl, pH 7.5, 60 mM MgCl_2_, 100 mM NaCl) and adjusted the volume to 70 µl with dH_2_O. The incubation continued for another 20 min and then the digested product was diluted with 210 µl of cold PBS, containing 5 mM magnesium acetate [28], [29].

### Affinity selection and in situ RT-PCR Recovery

Microtiter plate was coated at 4°C overnight with 100 µl of Avidin solution (0.066 µM in PBS), and then washed with PBS for three times and blocked with blocking buffer PBSB (PBS with 1% (w/v) BSA) for 2 h at room temperature (RT). After another PBS washing for three times, the plate was coated with 100 µl of Biotin-N-hydroxy-succinimide ester-sulfadimidine-ovalbumin (BNHS-SM_2_-OVA) at 4°C overnight. The coated plate was subsequently washed by ice-cold washing buffer (PBS with 0.05 % (v/v) Tween 20 and 5 mM magnesium acetate) for three times, and then placed on ice for at least 10 min. 100 µl the prepared TNT translation mixture containing the MRA ternary complexes was added to an antigen-coated well and incubated at 4°C for 2 h with gentle vibration. Followed three times wash with cold washing buffer and two times quick wash with ice-cold RNase-free water, in situ Single-Primer RT-PCR Recovery was performed in the plate wells carrying selected MRA complexes using PrimeScript^TM^ Reverse Transcriptase (TaKaRa). In brief, 12 µl of solution A mixture, containing 1 µl Primer RT-kz (10 µM), 2 µl dNTP (10 mM) and 9 µl RNase-free water, was added into each MRA-bound well. After incubating at 48°C for 5 min and at least 30 s on ice, the following reagents were added into each well: 200 units PrimeScript^TM^ Reverse Transcriptase, 20 units Rnase Inhibitor, 5 mM DTT, 4 µl 5×PrimeScript^TM^ buffer (250 mM Tris/HCl, pH 8.3, 15 mM MgCl_2_, 375 mM KCl) and adjusted the volume to 20 µl with RNase-free water. The plate was incubated at 42°C for 45 min followed by 5 min at 85°C. The mixture cooled to room temperature was then transfered to a fresh tube for subsequent single-primer PCR (SP-PCR) using primer KZ. A further PCR step was introduced to regenerate the full-length construct avoiding shortening of the DNA fragment, compared to the original fragment [29]. The primers used in the PCR amplification were shown in **Table 1**. The purified PCR products were used for the next round of ribosome display or cloned into *E. coli* HB2151 for expression.

### Affinity maturation

The selected scFvs of first cycle were subjected to PCR-based random mutagenesis by using the error-prone PCR and staggered extension process (StEP) shuffling in tandem, according to the protocols described by Cadwell [37] and Zhao[38] to generate the initial mutant ribosome library of anti-Sulfanilamides scFvs. EP-PCR reactions were carried out under the following conditions, hereafter referred to as standard: 10 ng of DNA template, 10×PCR buffer (Mg^2+^ Free, TaKaRa), 10 mM of each dNTP (dATP ∶ dTTP = dGTP ∶ dCTP = 1 ∶ 5), 10 µM of both primers (V_H_For*NcoI* and V_L_Rev*HindIII*), 25 mM MgCl_2_ and 2.5 U Taq DNA polymerase (TaKaRa) in 50 µl volume. PCR reactions were performed in an ABI thermocycler (Applied Biosystems Inc.) for 30 cycles: 1 min at 94°C, 1 min at 45°C and 2 min at 72°C, followed by 7 min extension at 72°C. Then the StEP shuffling was performed under the following conditions (5 min at 95°C, 80 cycles of 30 s at 94°C, and 5 s at 55°C, 5 min at 72°C), the StEP shuffling reactions contained (50 µl final volume): 10 ng of the DNA products of EP-PCR, 10×PCR buffer (Mg^2+^ Plus, TaKaRa), 10 mM of dNTP mixtures, 10 µM of both primers (V_H_For*NcoI* and V_L_Rev*HindIII*), 2.5 U Taq DNA polymerase (TaKaRa) and dH_2_O. The purified StEP shuffling products were used for the third round of ribosome display. The mutant repertoire was panned against target antigen as described above. One cycle of affinity maturation was performed.

### Cloning and expression of scFv

After four selections, the obtained scFv fragment was amplified with forward primer scFvFor*SfiI* with the *SfiI* restriction site (underlined) and reverse primer scFvRev*NotI* with the *NotI* restriction site (underlined). The primers used in the PCR amplification were shown in **Table 1**. The amplified product were digested with *SfiI* and *NotI*, then ligated with the vector pCANTAB5E by using a T4 DNA ligase (Promega, USA). The ligated products were transformed into *E.coli* HB2151 and the soluble scFv protein was expressed from each clone[39]. In brief, single colonies were grown in 5 ml of 2×YT medium with ampicillin (100 µg/ml) and glucose (0.1 % (w/v)) to an OD_600_ = 0.6 at 30°C/250 r.p.m, and induced by the addition of IPTG (final concentration 1 mM) overnight at 30°C/180 r.p.m. The cells were pelleted at 4°C/4000 r.p.m for 10 min, and re-suspended in 0.5 ml ice-cold 1×TES buffer (0.2 M Tris/HCl (pH 8.0), 0.5 mM EDTA, 0.5 M sucrose) and 0.75 ml ice-cold 1/5×TES buffer. After incubation on ice for 40 min, the cells were pelleted at 4°C/12000 r.p.m for 20 min, and the supernatant was retained as periplasmic extracts with the soluble scFvs. The expressed soluble scFvs were detected by using anti-E tag monoclonal antibody, since the pCANTAB5E vector contains an additional sequence encoding the E-tag.

### ELISA assays

To screen anti-SM_2_ specific scFvs, the expressed soluble scFvs were analyzed by indirect ELISA. Microtiter plates were coated with 100 µl of sulfadimidine-ovalbumin (SM_2_-OVA, 10 µg/ml in PBS) overnight at 4°C. Plates were washed with washing buffer and the blocking buffer (4 % (w/v) BSA in PBS, pH 7.4) was subsequently added at 37°C for 1 h with gentle shaking. Blocked plates were washed and 100 µl of periplasmic extracts diluted 1∶1 with PBSB were titrated followed by 1 h incubation at 37°C. Detection was performed with an **HRP**-labelled goat anti-E-tag antibody (1∶10000 dilution with blocking buffer). Plates were developed with TMB-detection-solution and read at OD450 nm.

### Immunoblot analysis of scFv expression

Periplasmic extracts from selected anti-sulfadimidine producing clones were subjected to SDS-PAGE on a 12 % polyacrylamide gel and Western blot. After SDS-PAGE, the gel was transferred onto a nitrocellulose membrane. The transblotted membrane was blocked for 1 h with a blocking buffer (4 % (w/v) BSA in PBS, pH 7.4) and then incubated with anti-E-tag antibody HRP conjugated (1∶2000) for 2 h at RT. 4-CN (4-chloro-1-naphthol, Sigma) was used as a peroxidase substrate to visualize the immunoreactivity.

### Sequence analysis

Plasmid DNA from anti-sulfadimidine producing clones was isolated from *E. coli* HB2151 using the QIAEX II gel extraction kit (QIAGEN). The scFv DNA was sequenced on both strands with the pCANTAB5E sequence primer set using an ABI Perkin Elmer 373A automated DNA sequencer.

## Supporting Information

Figure S1 Clones were isolated from the unselected antibody library. Each clone was expressed for producing soluble scFv, and the binding activity of each scFv and the anti-SM2 MAb (positive control) was determined by ELISA (in triplicate). The error bars represent the standard deviation.
(0.46 MB TIF)
